# Targeting Na_v_ Channels for Pain Relief: Structural Insights and Therapeutic Opportunities

**DOI:** 10.3390/ijms27031180

**Published:** 2026-01-23

**Authors:** Yuzhen Xie, Xiaoshuang Huang, Fangzhou Lu, Jian Huang

**Affiliations:** 1 Institute of Bio-Architecture and Bio-Interactions (IBABI), Shenzhen Medical Academy of Research and Translation (SMART), Shenzhen 518107, China; 2 Westlake University, Hangzhou 310030, China

**Keywords:** voltage-gated sodium channels, pain relief, pathological conditions, structural insights, mechanism of drug action

## Abstract

Pain is an unpleasant but essential sensory experience that serves as a protective mechanism, yet it can also manifest maladaptively in a wide range of pathological conditions. Current analgesic strategies rely heavily on opioid medications and non-steroidal anti-inflammatory drugs (NSAIDs); however, concerns regarding addiction, tolerance, and dose-limiting adverse effects highlight the urgent need for safer and more effective therapeutics. Voltage-gated sodium (Na_v_) channels, which govern the initiation and propagation of action potentials, have emerged as promising targets for mechanism-based analgesic development. In particular, the Na_v_1.7–Na_v_1.9 subtypes have attracted substantial interest owing to their enrichment in the peripheral nervous system—despite broader expression elsewhere—and their central roles in nociception, offering the potential for non-addictive, subtype-selective pain modulation. This review summarizes the physiological roles of these channels in nociception, examines how disease-associated mutations shape pain phenotypes, and highlights recent advances in drug discovery targeting Na_v_1.7 and Na_v_1.8. The recent FDA approval of VX-548 (suzetrigine), a first-in-class and highly selective Na_v_1.8 inhibitor, marks a major milestone that validates peripheral Na_v_ channels as clinically actionable targets for analgesia. We also discuss the remaining challenges and emerging opportunities in the pursuit of next-generation, mechanism-informed analgesics.

## 1. Introduction

Pain is a major global health issue arising from diverse pathological processes [1]. It can be categorized by duration (acute or chronic) and by underlying mechanisms, including nociceptive, neuropathic, nociplastic, and mixed pain, as well as secondary pain syndromes associated with specific diseases [2,3,4]. Although multiple classes of analgesics, such as opioids, non-steroidal anti-inflammatory drugs (NSAIDs), and various adjuvant agents, are clinically available, their utility is constrained by safety concerns, dependence liability, and insufficient efficacy in many patients [5,6]. These limitations highlight the urgent need for safer and more effective mechanism-based pain therapeutics.

Voltage-gated sodium (Na_v_) channels have emerged as promising molecular targets for developing non-addictive analgesics due to their crucial roles in the initiation and propagation of action potentials and in the regulation of nociceptor excitability [7]. Aberrent function or dysregulation of these channels can alter neuronal excitability and drive diverse disease phenotypes, including congenital insensitivity to pain and various forms of chronic pain [8]. In this review, we provide an overview of the functional and structural characteristics of the peripheral Na_v_1.7–Na_v_1.9 subtypes and summarize recent advances in the development of therapeutics targeting these channels for pain management.

## 2. Methodology

Search strategy: A comprehensive literature search was conducted in PubMed and Google Scholar using combinations of keywords including “voltage-gated sodium channel,” “pain,” “analgesic,” “structural basis,” “drug development,” and related synonyms. Searches were conducted up to November 2025.

Inclusion criteria: Studies were included if they (i) reported original experimental research, (ii) were directly relevant to pain mechanisms or analgesia involving Na_v_1.7–Na_v_1.9, and (iii) addressed the development, pharmacology, or structural characterization of therapeutics targeting these channels.

Exclusion criteria: Studies were excluded if they (i) were not published in English, (ii) were deemed irrelevant based on title and abstract screening, (iii) lacked assessable full-text data, or (iv) were not original research papers.

## 3. VGSC Family Overview

Na_v_ channels are crucial for neuronal excitability and the propagation of action potentials [9]. In 1952, Hodgkin and Huxley first demonstrated the role of Na_v_ channels in generating action potentials using the voltage clamp technique [10], laying the conceptual foundation for voltage-dependent sodium permeability well before the channel itself was characterized. Subsequent biochemical purification and molecular cloning efforts provided the first molecular insights into these channels [11]. Early structural insights were obtained using pore-blocking neurotoxins such as saxitoxin (STX) and tetrodotoxin (TTX) [12]. Catterall and colleagues later purified and functionally characterized Na_v_ channels from mammalian brain membranes, defining their pharmacology and gating properties [12,13]. Soon after, Noda and Numa isolated the cDNA encoding the Na_v_ channel from *Electrophorus electricus* electroplax, revealing its four-domain architecture and demonstrating that a single α-subunit is sufficient for sodium conductance upon expression in *Xenopus* oocytes [14,15].

**Figure 1:**
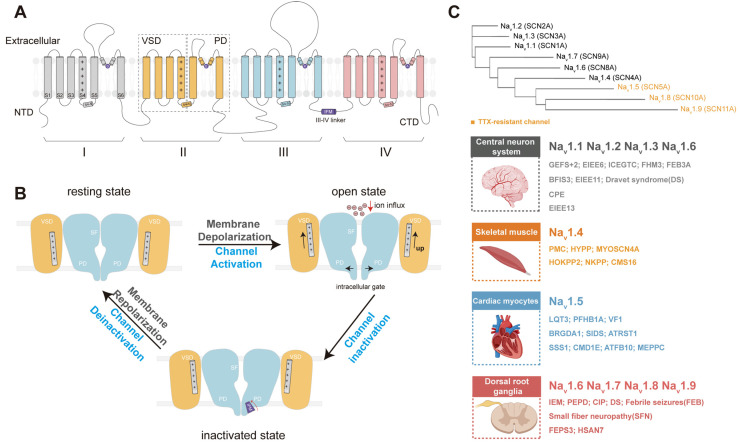
Topology, gating, and classification of human Na_v_ channels. (**A**) Schematic illustration showing the transmembrane topology of human Na_v_ channels. (**B**) Gating of Na_v_ channels. (**C**) Classification of the nine human Na_v_ isoforms. Top: Phylogenetic tree of human Na_v_ channels, with the three TTX-resistant subtypes (Na_v_1.5, Na_v_1.8, and Na_v_1.9) highlighted in orange. Bottom: Tissue distribution of human Na_v_ channels and representative channelopathies caused by functional disorders of these channels. Na_v_1.1 is associated with GEFS+2, EIEE6, ICEGTC, FHM3, and FEB3A; Na_v_1.2 with BFIS3, EIEE11, and DS; Na_v_1.3 with CPE; Na_v_1.4 with multiple neuromuscular disorders including PMC, HOKPP2, HYPP, NKPP, MYOSCN4A, and CMS16; Na_v_1.5 with PFHB1A, LQT3, BRGDA1, SSS1, VF1, SIDS, ATRST1, CMD1E, ATFB10, and MEPPC; Na_v_1.6 with EIEE13; Na_v_1.7 with IEM, PEPD, CIP, DS, SFN, and FEB; Na_v_1.8 with SFN; and Na_v_1.9 with FEPS3 and HSAN7. See the Abbreviations section for full disease names.

Na_v_ channels display a fourfold (pseudo-)symmetry around a central axis perpendicular to the membrane [16]. The α-subunit of eukaryotic Na_v_ channels is a single polypeptide comprising four homologous repeats, each containing six transmembrane segments (S1–S6) (Figure 1A). S1–S4 in each repeat form voltage-sensing domains (VSDs), whereas S5–S6 from all repeats assemble into the central pore domain (PD) [17]. Most Na_v_ channels adopt a domain-swapped architecture, in which each VSD interacts with the pore of the adjacent domain in a clockwise arrangement when viewed from the extracellular side [16]. Ion selectivity of the Na_v_1 family is conferred by the highly conserved Asp-Glu-Lys-Ala (DEKA) selectivity filter, with one residue contributed by each repeat [18].

Na_v_ channels transition among three major voltage-dependent conformational states: resting, activated, and inactivated states (Figure 1B). Upon depolarization, outward movement of the S4 helices initiates pore opening and drives the rapid influx of Na^+^ ions. Within milliseconds, channels undergo fast inactivation mediated by the conserved Ile-Phe-Met (IFM) motif in the III–IV linker. The IFM motif binds to a hydrophobic receptor site between repeats III and IV, promoting S6 helix constriction and preventing further ion conduction [17]. Impaired or incomplete inactivation results in a persistent sodium current, which contributes to subthreshold excitability and supports repetitive firing in nociceptors and other excitable cells [19].

In humans, nine α-subunit isoforms (Na_v_1.1 to Na_v_1.9) and four auxiliary β-subunits (β1 to β4) constitute the Na_v_ channel family. β subunits modulate channel gating, trafficking, and membrane localization. A classical framework for distinguishing Na_v_ isoforms is their sensitivity to TTX, which is currently under clinical evaluation for cancer-related and chemotherapy-induced neuropathic pain [20]. Na_v_1.5, Na_v_1.8, and Na_v_1.9 are TTX-resistant isoforms, and accumulating evidence implicates them in the development and maintenance of neuropathic pain [16,20].

Na_v_ isoforms exhibit highly distinct tissue-specific expression patterns. Na_v_1.1–Na_v_1.3 are predominantly expressed in the central nervous system (CNS), where they regulate neuronal excitability, and mutations in their corresponding genes are associated with epilepsy and other neurodevelopmental disorders [21,22]. Na_v_1.4 is the principal isoform in skeletal muscle, whereas Na_v_1.5 is the major isoform in cardiac myocytes. Na_v_1.6 is broadly distributed across both the CNS and peripheral nervous system (PNS). In contrast, Na_v_1.7–Na_v_1.9 are preferentially expressed in peripheral sensory neurons, particularly nociceptors (Figure 1C) [21]. Extensive evidence indicates that these peripheral isoforms are crucial for transmitting nociceptive signals from the periphery to the CNS, positioning them as key molecular targets for analgesic development.

## 4. Overview of Peripheral VGSC Isoforms and Nociception

Nociceptors are primary sensory neurons that detect noxious mechanical, thermal, or chemical stimuli and convey pain information to the CNS [2]. They are widely distributed throughout the body, including skin, joints, and organ walls. Nociception involves three key stages: transduction, where peripheral terminals of nociceptors convert harmful stimuli into electrical signals; transmission, during which action potentials propagate along the axon from the peripheral terminals toward the central terminals; and synaptic transmission, in which neurotransmitters are released in the dorsal horn of the spinal cord to activate CNS neurons. Na_v_ channels are essential to each of these processes by mediating action potential initiation and propagation, thereby enabling nociceptors to encode and transmit pain information with high fidelity [23].

Nociceptors comprise two major fiber types: large myelinated Aδ fibers, which transmit sharp, well-localized pain, and unmyelinated C fibers, which convey slower, burning, or aching sensations. Single-cell RT-PCR analyses have demonstrated that the three peripheral Na_v_ channel isoforms, Na_v_1.7–Na_v_1.9, are predominantly expressed in C fiber sensory neurons [24]. These channels display distinct yet complementary biophysical properties that collectively shape nociceptor excitability [25]. Accordingly, genetic mutations or altered expression of these channels can give rise to a range of pain disorders, underscoring their potential as therapeutic targets [26].

In addition to its prominent expression in C fibers, Na_v_1.7 is robustly localized to the nodes of Ranvier in a subpopulation of myelinated Aδ fibers [27], positioning it to influence both action potential initiation and propagation. Consistent with this distribution, Na_v_1.7 exhibits rapid activation and inactivation kinetics together with a slow onset of inactivation, enabling it to act as a threshold channel and facilitate action potential initiation in nociceptors [28,29]. By contrast, Na_v_1.8 activates at more depolarized membrane potentials and exhibits slower inactivation, contributing substantially to the action potential upstroke and supporting repetitive firing during sustained stimuli [30,31]. Na_v_1.9 produces a persistent, subthreshold sodium current that modulates resting membrane potential and overall neuronal excitability [32]. Together, these channels orchestrate the initiation and propagation of nociceptive signals in peripheral sensory neurons.

The following section examines the physiological and pathological roles of Na_v_1.7–Na_v_1.9, with particular emphasis on the functional consequences of disease-associated mutations, recent progress in isoform-selective drug development, and emerging therapeutic strategies.

## 5. Na_v_1.7: Pathophysiological Role and Structural Basis

### 5.1. Physiological Function and Disease Mutations

**Figure 2:**
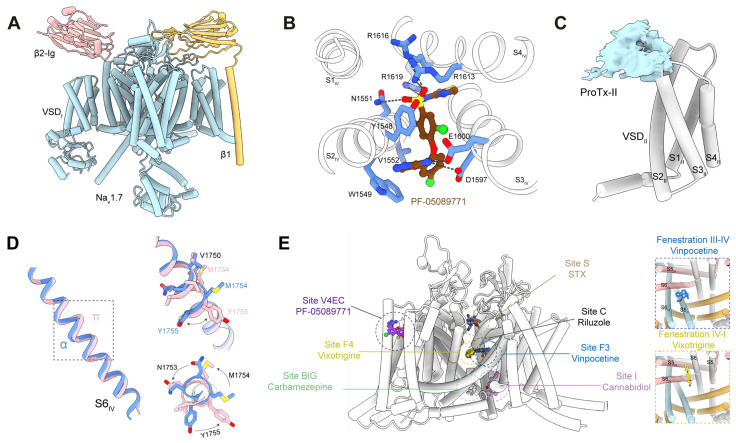
Structural features of Na_v_1.7 and binding modes of representative inhibitors. (**A**) Overall structure of Na_v_1.7 in complex with β1 and β2 subunits (PDB: 7W9T). (**B**) Coordination of PF-05089771 in Na_v_1.7, with key interacting residues shown as blue sticks (PDB: 8I5G). Hydrogen bonds are indicated by gray dashed lines. (**C**) Cryo-EM density of the toxin ProTx-II positioned above VSD_II_. The toxin density is shown in cyan, and VSD_II_ is shown in grey cylinders. (**D**) Structural comparison of the toxin-bound and apo Na_v_1.7 showing an α to π helical transition in S6_IV_ (α helix PDB: 7W9T; π helix PDB: 7W9P). The right panel highlights residues that undergo notable rotational changes. (**E**) Representative Na_v_1.7 inhibitors mapped onto the structural model to illustrate the major pharmacological binding sites.

Na_v_1.7, encoded by *SCN9A*, is broadly expressed across the PNS and CNS, with particularly high enrichment in small-diameter dorsal root ganglia (DRG) neurons and sympathetic ganglia (Figure 2A) [27]. Within nociceptive pathways, Na_v_1.7 operates as a threshold channel that amplifies subthreshold depolarizations and supports action potential initiation at both peripheral and central terminals [33]. Dysregulation of Na_v_1.7 can therefore shift neuronal excitability into pathological ranges.

The clinical relevance of Na_v_1.7 is underscored by the striking genotype-phenotype correlations observed across inherited channelopathies. Gain-of-function (GOF) mutations drive hyperexcitability and give rise to three major syndromes. In inherited erythromelalgia (IEM), patients experience burning pain in the extremities, often associated with hyperpolarizing shifts in channel activation [29]. Paroxysmal extreme pain disorder (PEPD) is characterized by severe rectal, ocular, and submandibular pain and frequently results from impaired fast inactivation, often linked to mutations in the III-IV linker [34]. Small fiber neuropathy (SFN) presents with burning pain and autonomic dysfunction due to selective damage to unmyelinated C fibers and thinly myelinated Aδ fibers [35,36]. Na_v_1.7 variants associated with SFN typically impair slow inactivation, shift steady-state inactivation toward depolarized potentials, or generate enhanced resurgent currents, providing biophysical features that distinguish SFN from IEM and PEPD [35,37,38].

In contrast, loss-of-function (LOF) mutations in Na_v_1.7 produce a diametrically opposite phenotype. Biallelic LOF variants cause congenital insensitivity to pain (CIP), a condition characterized by a complete absence of pain perception despite preserved tactile sensation [23,39]. Electrophysiological studies demonstrate that CIP-associated nonsense mutations abolish Na_v_1.7–mediated currents, thereby eliminating its contribution to spike initiation in nociceptors [40]. However, studies in Na_v_1.7–null models have shown that the excitability of peripheral sensory neurons is preserved, suggesting that factors beyond impaired action potential generation may contribute to the analgesic phenotype. Potential explanations include alterations in opioid signaling and compensatory upregulation of interacting proteins such as other Na_v_ subtypes and auxiliary subunits [41,42]. These findings imply that analgesia associated with Na_v_1.7 loss of function cannot be explained solely by reduced peripheral excitability. Instead, downstream pathways linked to Na_v_1.7 function, including opioid signaling, may represent alternative targets for pain modulation [43].

These GOF and LOF phenotypes establish Na_v_1.7 as a key molecular determinant of human pain sensitivity. A review published in 2020 mapped known pathogenic variants onto the Na_v_1.7 structure, providing a valuable framework linking structural elements to biophysical and clinical phenotypes [44].

### 5.2. Channel Modulators

Two main therapeutic strategies have been explored for targeting Na_v_1.7: pore blockers and gating-modifier toxins (GMTs). State-dependent pore blockers bind within the central cavity and preferentially interact with a specific functional state [45]. Despite the clinical success of state-dependent Na_v_ channel inhibitors in epilepsy, analogous strategies for pain relief have faced challenges, primarily due to the high conservation of pore residues across Na_v_ isoforms, which limits subtype selectivity [46]. Although clinical candidates such as PF-05089771 (Pfizer) and vixotrigine (Biogen) achieved high Na_v_1.7 selectivity in vitro, both were ultimately discontinued due to the lack of meaningful analgesic efficacy in clinical trials, with only modest pain reduction observed (Figure 2B and Table 1). More broadly, many Na_v_1.7–selective inhibitors have failed in clinical development owing to a combination of factors, including off-target effects leading to adverse events such as nausea and vomiting, inadequate potency in human nociceptors, and poor translation from rodent to human pain phenotypes [30]. Recent efforts have shifted toward identifying small molecules that exploit structural divergence in VSDs and cytoplasmic linkers to improve isoform selectivity [45,47].

GMTs, primarily venom-derived peptides, target VSDs and stabilize specific channel states. Depending on their mode of action, GMTs can either suppress hyperexcitability or trigger sustained channel activation [45]. Although many GMTs exhibit exceptional subtype selectivity for Na_v_1.7, their in vivo efficacy remains variable. For example, protoxin II (ProTx-II), isolated from the tarantula *Thrixopelma pruriens*, displays at least 100-fold selectivity for Na_v_1.7 over other isoforms but shows limited analgesic activity in vivo, likely due to restricted blood–brain barrier penetration and rapid systemic clearance (Figure 2C) [45].

Broad-spectrum Na_v_ channel inhibitors, including lidocaine, mexiletine, and carbamazepine, remain clinically relevant but exhibit weak inhibitory potency and poor subtype selectivity [46,47]. While their precise mechanisms of action are not fully resolved, their proven efficacy in both preclinical and clinical settings supports the notion that appropriately tuned modulation of Na_v_ activity can yield effective analgesia without necessitating complete channel blockade [46].

Collectively, these pharmacological and genetic insights illustrate how even subtle alterations in Na_v_1.7 function can profoundly influence nociceptor excitability and pain perception. The limited clinical success of existing inhibitors underscores the need for next-generation, subtype-selective modulators guided by structural and biophysical understanding. Recent advances in cryo-electron microscopy (cryo-EM) and computational modeling have begun to elucidate the molecular determinants underlying Na_v_1.7 gating, pharmacology, and disease-associated mutations, offering a powerful framework for structure-guided drug discovery in pain therapeutics.

### 5.3. Structural Insights into Drug Targeting and Remaining Challenges

The first cryo-EM structure of human Na_v_1.7, a toxin-bound E406K variant, was reported by Shen et al. (2019), providing the foundational model of the channel’s architecture [100]. Subsequent studies improved the resolution of the wild-type channel to 2.2 Å, allowing detailed visualization of gating helices, voltage sensors, and drug-binding pockets (Figure 2A) [101]. Comparative analysis between toxin-bound and apo structures revealed a gating-linked α→π helical transition in S6_IV_, which reshapes the pore geometry and influences the conformational states accessible to small-molecule modulators (Figure 2D).

These structural advances have delineated multiple ligand-binding sites on Na_v_1.7, including: Site E, the extracellular peptide toxin interface; Site S, the selectivity filter recognized by guanidinium toxins such as TTX/STX; Site C, the central cavity for diverse small molecules; Site F, lipid-facing fenestrations accessed by compounds like vixotrigine; Site G, the intracellular gate that modulates state-dependent block; Site BIG, a pocket beneath the intracellular gate; and Site I, an inactivation-motif docking site targeted by cannabidiol (Figure 2E) [46,47]. The integration of these high-resolution structural maps with disease-related mutations enables the 3D annotation of pharmacologically relevant residues and supports structure-guided drug design. Future cryo-EM structures of Na_v_1.7 variants associated with pain disorders may further reveal how pathogenic substitutions perturb gating properties, destabilize specific functional states, or alter ligand selectivity, thereby refining opportunities for selective modulator design [47,100,102].

Despite strong genetic evidence supporting Na_v_1.7 as a pain target, efforts to develop clinically effective analgesics have repeatedly fallen short. Small-molecule inhibitors such as XEN402 (Funapide) exhibited state-dependent block and promising in vitro selectivity but showed limited efficacy in clinical trials, likely due to pharmacokinetic limitations, and development was discontinued in 2020 [67]. Moreover, Na_v_1.7 expression extends beyond nociceptive neurons, and it is also found in olfactory neurons and pancreatic β-cells, where it contributes to olfactory signaling and glucose homeostasis [103]. Consequently, systemic inhibition risks metabolic and sensory side effects that complicate dose optimization. Achieving the right therapeutic window is further constrained by the physiological role of Na_v_1.7, as human genetic data suggest that at least ~50% inhibition can produce analgesia without compromising protective pain sensation, whereas complete blockade mimics congenital insensitivity to pain [30].

**Figure 3:**
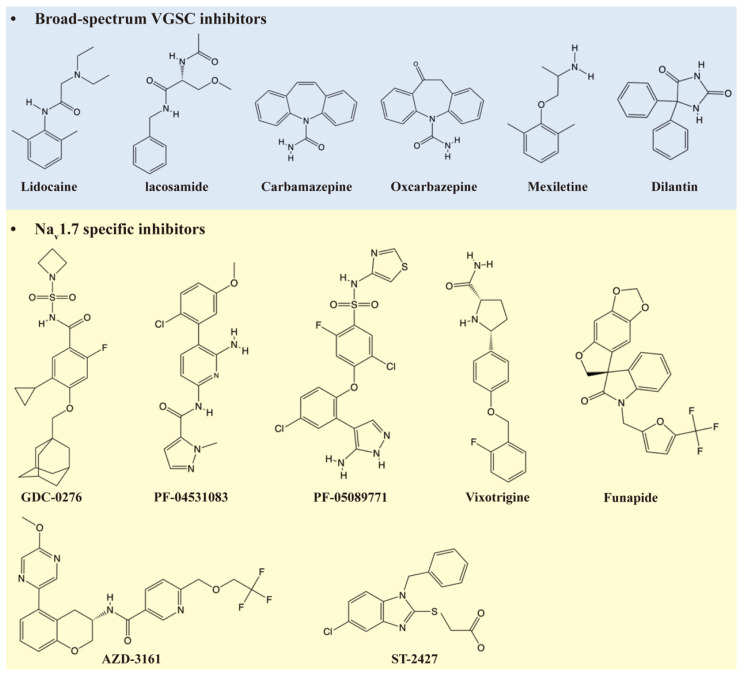
Chemical structures of representative broad-spectrum VGSC inhibitors and Na_v_1.7–selective inhibitors.

Current efforts are moving beyond conventional pore blockers toward state-dependent gating modifiers and structure-guided allosteric inhibitors that leverage conformational plasticity revealed by recent cryo-EM studies (Figure 3) [46,47]. Alternative strategies such as gene therapy and monoclonal antibodies targeting voltage-sensing domains are emerging as promising directions for achieving isoform-selective modulation [45,104]. A major barrier remains the translation gap between preclinical models and human pain perception, complicated by differences in pain etiology (neuropathic vs. chronic), species-specific gating properties, and limited demographic diversity in clinical trials [105]. Despite this, as observed in CIP, loss of Na_v_1.7 expression might affect the endogenous opioid system, providing a possible explanation for the limited efficacy of selective Na_v_1.7 modulators and motivating alternative strategies such as targeting downstream effectors or employing synergistic therapeutic approaches [106]. Additionally, Na_v_1.7 is also dynamically regulated by inflammatory signaling. Mediators such as protein kinase C (PKC) can elevate Na_v_1.7 expression and alter its gating properties in nociceptive neurons during chronic inflammation [25,29]. Such regulation highlights Na_v_1.7 as an important mediator of inflammatory and neuropathic pain pathways.

Looking forward, the integration of structural biology with molecular dynamics simulations and large-scale computational screening is poised to accelerate the discovery of selective and non-addictive Na_v_1.7 modulators. Yet, despite its compelling genetic validation, converting structural insights into effective clinical therapies remains a major challenge that requires multidisciplinary collaboration across structural biology, pharmacology, and neurophysiology.

## 6. Na_v_1.8

### 6.1. Physiological Function, Disease Mutations, and Channel Modulators

*SCN10A* encodes Na_v_1.8, which is expressed predominantly in peripheral sensory neurons, particularly DRG neurons, with relatively low expression in most other tissues and regions [107]. Na_v_1.8 generates a TTX-resistant current and is considered a major contributor to the rising phase of the action potential in nociceptive neurons under depolarized conditions, as well as to repetitive firing during nociceptive signaling [31]. Additionally, previous studies have shown that human Na_v_1.8 exhibits a substantial persistent current that contributes to the regulation of repetitive firing [108]. These electrophysiological features establish Na_v_1.8 as an important regulator in chronic and neuropathic pain [30,31].

Genetic analyses have identified several GOF mutations in Na_v_1.8 that enhance neuronal excitability, while LOF variants have not been clearly documented. However, some polymorphisms are associated with reduced pain sensitivity [9,23]. GOF mutations generally accelerate recovery from inactivation or shift activation thresholds in the hyperexcitable direction, promoting increased firing of nociceptive neurons. These functional alterations are linked to pain syndromes such as SFN and other neuropathic conditions [109,110,111]. Beyond genetic variants, inflammatory mediators and tissue injury can also modulate Na_v_1.8 expression and gating properties, providing alternative mechanisms through which the channel contributes to pathological pain [29].

Na_v_1.8 is also expressed in visceral sensory pathways, with emerging evidence implicating its involvement in visceral pain disorders, including inflammatory bowel disease (IBD). Certain Na_v_1.8 variants correlate with altered visceral pain sensitivity, though the mechanistic basis remains to be clarified [112,113]. These findings highlight the broad physiological relevance of Na_v_1.8 across peripheral and visceral nociceptive pathways.

Therapeutic efforts targeting Na_v_1.8 have faced challenges due to the difficulty of achieving selective modulation with minimal side effects. A major breakthrough came with VX-548 (suzetrigine), optimized from VX-150, a highly selective Na_v_1.8 inhibitor with remarkable isoform specificity (>30,000-fold) and favorable pharmacokinetic properties. Clinical studies have demonstrated its efficacy in acute postoperative pain, providing opioid-comparable analgesia without addictive potential or significant CNS adverse effects [114]. 

Using 10 nM VX-548 (IC_50_ < 1 nM), Stewart et al. demonstrated that VX-548 significantly reduced both the peak amplitude and the shoulder of action potentials in DRG neurons, consistent with previous findings that Na_v_1.8 contributes to the later rising phase of the action potential [115]. They further observed that repetitive firing during sustained depolarization was attenuated, but not fully eliminated, by VX-548. This incomplete suppression may reflect the high expression of Na_v_1.8 in DRG neurons or compensatory activity mediated by Na_v_1.7 [115]. Collectively, these findings offer a mechanistic basis for the limited analgesic efficacy observed in a subset of clinical cases. 

This FDA approval of VX-548 marks a significant milestone in Na_v_ channel-targeted analgesia, positioning Na_v_1.8 as a clinically validated molecular target for pain management. While current evidence supports its role primarily in acute and neuropathic pain, further investigations are needed to explore efficacy across diverse pain conditions and patient populations, including special clinical settings such as pregnancy [6].

In summary, the unique biophysical properties and critical roles of Na_v_1.8 in nociceptive signaling underscore its importance in pain pathophysiology and therapeutic intervention. The success of selective Na_v_1.8 inhibitors opens a new avenue for developing safer, non-opioid analgesics tailored to modulate peripheral neuronal excitability in various pain disorders.

### 6.2. Structural Insights into Drug Targeting

**Figure 4:**
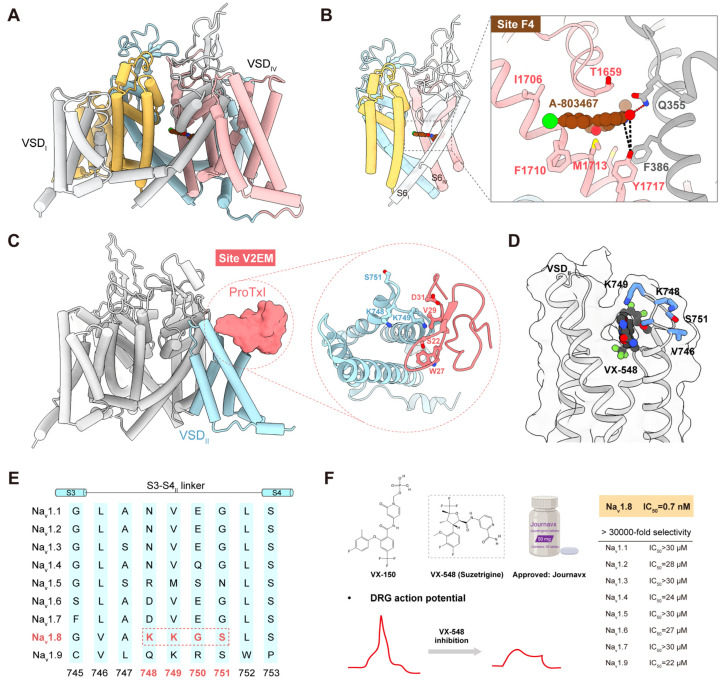
Structural and functional features of Na_v_1.8 and its inhibitors. (**A**) Overall structure of Na_v_1.8 in complex with the selective inhibitor A-803467 (PDB: 7WE4). (**B**) A-803467 clenches S6_IV_ of Na_v_1.8 beneath the selectivity filter. Inset: Coordination of A-803467. Direct and water-mediated hydrogen bonds (H-bonds) are indicated by red and black dashed lines, respectively. (**C**) ProTxI binds above VSD_II_ of Na_v_1.8, with VSD_II_ colored in cyan and ProTxI shown in light-coral surface representation. Inset: Detailed coordination between Na_v_1.8 and ProTxI (PDB: 9DBN). (**D**) Predicted binding pose of the clinical inhibitor VX-548, derived from Glide docking in Maestro using the Na_v_1.8 structure (PDB: 7WE4). Residues in the S3-4 loop adjacent to VX-548 are highlighted as blue sticks. (**E**) Sequence alignment of the VSD_II_ S3-4 linker. Residues highlighted by the red dashed box are experimentally verified to influence the inhibitory potency of VX-548 on Na_v_1.8. (**F**) Top left: Chemical structures of VX-150 and VX-548. Bottom left: Schematic representation of action potentials in DRG neurons before and after VX-548 inhibition, showing that VX-548 markedly decreases both the peak amplitude and the shoulder of action potentials. Right: IC_50_ values of VX-548 across human Na_v_ isoforms, measured by patch-clamp recordings. IC_50_ values were determined based on inhibition of the peak currents (Ref. [50]).

The first high-resolution cryo-EM structures of human Na_v_1.8 in both apo and A-803467-bound state were resolved in 2022 by Huang et al., with overall resolutions of 2.7–3.2 Å (Figure 4A). This study revealed that variations at the VSD_I_–PD interface contribute to the high voltage requirement for activation unique to Na_v_1.8 and identified key residues governing the selectivity of A-803467 (Figure 4B). In addition, the authors proposed a potential functional role for the extracellular loops, suggesting new opportunities for future drug design [116]. Together, these findings provide important structural insights for structure-guided discovery of Na_v_1.8-targeted analgesics.

Building on these results, Wang et al. (2024) systematically mapped the druggable sites on Na_v_1.8 using structural and computational analyses [114]. Four major sites were highlighted: Site E, the extracellular loop region above the pore domain targeted by conotoxins; Site C, the central pore cavity accommodating small-molecule inhibitors; Site V2E, an extracellular pocket in VSD_II_ that recognizes both small molecules and peptide toxins; and Site BIG, a broader intracellular interface, serves as a binding site for non-selective analgesics [114,117].

More recently, Neumann et al. (2025) resolved the cryo-EM structure of human Na_v_1.8 bound to Protoxin-I, a tarantula-derived peptide that shifts channel activation toward more depolarized potentials (Figure 4C) [118]. This study not only elucidated the molecular interaction between Protoxin-I and VSD_II_ but also emphasized the value of venom-derived peptides as templates for selective modulator development.

A similar binding site has been proposed for VX-548. Although the structure of VX-548-bound Na_v_1.8 has not yet been resolved, its binding site has been inferred through domain-swap experiments between Na_v_1.8 and Na_v_1.2. VX-548 is thought to stabilize Na_v_1.8 in the closed state; however, sufficient depolarization can promote dissociation of the compound [119]. These studies suggest that the KKGS sequence within VSD_II_ confers isoform selectivity, suggesting a mechanism that offers a blueprint for designing future Na_v_1.8-selective modulators (Figure 4D–F) [48].

In summary, advances in cryo-EM-based structural biology have substantially accelerated progress in Na_v_1.8 research, enabling visualization of inhibitor binding sites and conformational dynamics at near-atomic resolution. While bridging the gap between preclinical efficacy and clinical translation remains a challenge, the FDA approval of VX-548 (suzetrigine) underscores the therapeutic potential of Na_v_1.8 as a validated target for pain relief and highlights the power of structure-guided drug discovery in developing safer, non-opioid analgesics.

## 7. Na_v_1.9

### 7.1. Physiological Function, Disease Mutations, and Inflammatory Pain

Compared with Na_v_1.7 and Na_v_1.8, both of which have been extensively studied as therapeutic targets, research on Na_v_1.9 has progressed more slowly, largely due to difficulties in isolating the channel from native neurons and establishing reliable heterologous expression systems. Recent advances, including the robust expression platform reported by Theys et al., have begun to overcome these technical barriers and allow more systematic functional and pharmacological characterization of this channel [32].

Na_v_1.9, encoded by *SCN11A*, is predominantly expressed in nociceptive neurons of the PNS, especially DRG neurons, where it contributes to pain signaling [120,121]. Although several studies mention the expression of Na_v_1.9 in the CNS (such as the spinal cord, hypothalamus), its precise cellular and subcellular distribution remains incompletely defined [122,123]. This channel activates at relatively negative membrane potentials and produces a TTX-resistant sodium current with ultraslow gating kinetics, resulting in a persistent current following activation [8]. These properties generate a substantial window current within the physiological voltage range, allowing Na_v_1.9 to amplify weak depolarizing inputs and maintain subthreshold depolarization [8,32]. Rather than driving the action potential upstroke, Na_v_1.9 regulates excitability by lowering the action potential threshold and facilitating repetitive firing in nociceptors [124].

*SCN11A* mutations are associated with clinical phenotypes ranging from CIP to episodic pain syndromes, SFN, and other painful neuropathies [125]. Unlike Na_v_1.7, where GOF mutations consistently cause hyperexcitability and LOF mutations cause hypoexcitability, Na_v_1.9 mutations present a more complex relationship between channel activity and clinical phenotype. Many pathogenic variants induce hyperpolarizing shifts in activation and expand the window current, consistent with GOF mechanisms. Yet patients may paradoxically experience pain insensitivity [126]. This counterintuitive phenotype can potentially be explained by a U-shaped model in which moderate depolarization increases neuronal firing, but excessive depolarization inactivates other peripheral Na_v_ channels, prevents action potential generation, and produces hypoexcitability [125,126].

Beyond genetic variants, Na_v_1.9 is strongly regulated by inflammatory mediators, positioning the channel as a key contributor to inflammatory pain. Molecules such as Bradykinin, ATP, histamine, prostaglandin-E2, and norepinephrine potentiate Na_v_1.9 activity [127,128]. These signaling pathways act synergistically through G-protein-dependent mechanisms, increasing open probability and mean open time, and may engage downstream kinases such as PKC to further influence gating [129]. These mechanisms amplify Na_v_1.9 function during inflammation, promoting persistent nociceptor hyperexcitability.

### 7.2. Development of Channel Modulators Targeting Na_v_1.9

Despite strong evidence supporting the role of Na_v_1.9 in nociception and inflammatory pain, the development of subtype-specific modulators has proven exceptionally challenging. Major obstacles include the channel’s complex and incompletely defined physiological functions, longstanding difficulties in achieving robust recombinant expression. To date, no high-resolution structural information is available for Na_v_1.9. Consequently, structural and functional insights have largely relied on homology modeling based on other Na_v_ channel isoforms, as well as the use of chimeric constructs to study gating properties and disease-associated dysfunction [32]. As a result, progress in identifying Na_v_1.9-selective small molecules has been slow. Most reported compounds that modulate Na_v_1.9 activity lack subtype specificity and often exhibit concurrent inhibition of Na_v_1.7 and Na_v_1.8, leading to broad-spectrum effects across multiple pain modalities (Table 1) [68].

In the future, alternative therapeutic strategies may emerge by targeting the regulatory proteins and signaling pathways that modulate Na_v_1.9 activity under inflammatory conditions. Intervening in these upstream or downstream effectors could provide an alternative route for achieving functional specificity even in the absence of direct, selective Na_v_1.9 inhibitors.

## 8. Future Directions and Limitations

### 8.1. Challenges and Opportunities

**Figure 5:**
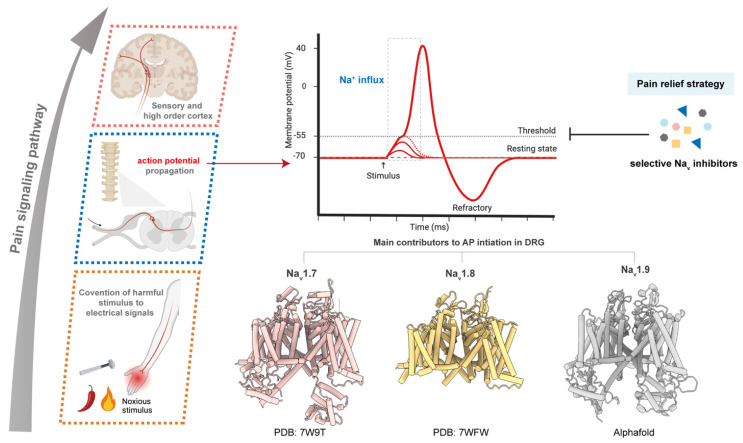
Molecular and functional roles of Na_v_1.7–Na_v_1.9 in pain signaling and their implications for peripheral Na_v_-targeted analgesic development.

Earlier efforts on Na_v_1.7 ultimately yielded disappointing outcomes in clinical trials, and the physiological and pharmacological properties of Na_v_1.9 remain only partially understood. Consequently, attention has increasingly shifted toward Na_v_1.8. The recent approval of VX-548 is particularly encouraging, as its development from gene identification and functional characterization to recognition as a validated drug target illustrates a successful, decades-long trajectory that provides a framework for developing novel, effective, and durable non-opioid analgesics (Figure 5).

Despite this progress, many investigational Na_v_-targeting drugs have failed during development. One major challenge is the high degree of structural conservation among Na_v_ channel subtypes, particularly within the pore domain, which limits isoform selectivity and increases the risk of off-target effects [100,116]. The broad distribution of Na_v_1.7–1.9 might hinder selectivity, which can also cause side effects. In addition, mechanistic insights from Na_v_1.7 loss-of-function models indicate that inhibition of Na_v_1.7 alone may be insufficient to produce robust analgesia, as other downstream signaling pathways might also be involved in pain insensitivity. Although recent advances in experiments have substantially improved our understanding of pain mechanisms and drug actions, a major gap remains between in vitro observations and clinical performance. A key challenge is the complexity of the in vivo environment, where Na_v_ channel dysfunction is influenced not only by changes in channel gating but also by transcriptional regulation, membrane trafficking, auxiliary protein interactions, and post-translational modifications [130]. A deeper characterization of these regulators may reveal new intervention points within peripheral pain pathways and guide future target discovery. External factors such as diet, concomitant medications, and metabolic variability can also affect drug efficacy and should be systematically addressed in clinical trial design. Ensuring demographic diversity across age, sex, race, and comorbid conditions is essential for accurately evaluating both analgesic responses and safety profiles.

The clinical success of VX-548 provides compelling proof of concept for non-opioid analgesics in the treatment of acute pain, demonstrating that effective analgesia can be achieved through peripheral Na_v_ inhibition while minimizing opioid-associated adverse effects. Peripherally restricted and subtype-selective Na_v_ inhibitors are particularly well suited for acute pain management, where rapid onset and short-term efficacy are desired, although long-term safety and effectiveness warrant careful evaluation [131].

By contrast, chronic pain presents distinct therapeutic challenges that require sustained modulation of nociceptor activity. Rather than targeting a single Na_v_ subtype, further drug development may benefit from broader or combinatorial modulation of Na_v_1.7–Na_v_1.9 to more effectively suppress pathological excitability while minimizing CNS involvement. In addition, combination therapies that integrate Na_v_-targeting agents with opioids or downstream modulators of nociceptor signaling may enhance analgesic efficacy at lower doses, thereby reducing the risk of central adverse effects such as tolerance and dependence [132]. Emerging strategies, including gene therapy strategies to selectively silence Na_v_1.8-expressing DRG neurons and peptide-based therapeutics derived from venom toxins, represent promising avenues for achieving durable and mechanism-informed analgesia [70,133,134].

Together, these advances highlight both the challenges and substantial opportunities that lie ahead. As our understanding of peripheral Na_v_ channel regulation deepens and the intervention of novel therapeutics, next-generation targeted analgesics may hopefully bridge the longstanding gap between mechanistic insights and effective clinical pain relief.

### 8.2. Limitations of This Review

This review focuses on Na_v_1.7–Na_v_1.9 as specific molecular targets for pain relief, with an emphasis on improving drug efficacy and long-term sustainability. General analgesics that modulate Na_v_ channels in a non-selective manner are acknowledged as alternative therapeutics, whereas their MOA and signaling pathways fall beyond the scope of this review. Given the extensive efforts in this field, the most recent drug candidates or unpublished developments may not be fully captured. In addition, emerging therapeutics, including peptide-based and nucleic acid-based sodium channel modulators, are not discussed in detail and warrant further consideration in future studies.

## 9. Conclusions

Peripheral voltage-gated sodium channels Na_v_1.7–Na_v_1.9 are central regulators of nociception and represent critical molecular targets for the development of non-opioid analgesics, as demonstrated by converging genetic, electrophysiological and pharmacological studies. The recent clinical approval of the Na_v_1.8-selective inhibitor VX-548 for acute pain provides clinical validation for peripheral Na_v_ targeting and highlights the therapeutic potential of Na_v_1.7–Na_v_1.9 in pain management. Advances in cryo-EM have enabled high-resolution structural characterization of Na_v_1.7 and Na_v_1.8, supporting structure-guided drug discovery. Future progress in Na_v_-targeted analgesics will benefit from integrating structural, genetic, and physiological insights with a deeper understanding of the downstream pathways and compensatory mechanisms triggered by Na_v_ channel dysfunction.

## Figures and Tables

**Table 1:** Development of next-generation analgesics targeting the Na_v_1 family in advanced clinical trials.

Drugs	Companies	Targets	Mechanisms of Action	Clinical Development	Comments	Ref.
**Selective Inhibitors**						
Suzetrigine/Journavx (VX-548)	Vertex Pharmaceuticals	Na_v_1.8	Highly selective Na_v_1.8 blockers	Phase 3 COMPLETED (NDA submitted)	First-in-class Na_v_1.8 selective inhibitor. Demonstrated significant pain reduction vs. placebo in post-surgical acute pain. Non-opioid with potential for chronic pain applications	[48,49,50]
VX-993	Vertex Pharmaceuticals	Na_v_1.8	Next-gen Na_v_1.8 selective blocker	Phase 2 failed	Vertex is developing additional Na_v_1.8 candidates (e.g., VX-993) as follow-ons to VX-548	[51]
JMKX-000623	Shanghai Jemincare	Na_v_1.8	Selective Na_v_1.8 blocker	Phase 2	Evaluated the efficacy and safety of JMKX-000623 in participants with diabetic peripheral neuropathic pain	[51,52]
LTG-305	Latigo Bio	Na_v_1.8	-	Phase 1	LTG-305, a potential best-in-class non-opioid therapeutic candidate for the treatment of chronic pain	[51,53]
HBW-004285	Hyperway	Na_v_1.8	Na_v_1.8 blocker	Phase 2	Indication for pain, less information about clinical efficacy	[51]
STC-004	SiteOne (Lilly)	Na_v_1.8	-	Phase 1 COMPLETED	A novel inhibitor in development for the non-opioid treatment of pain	[54]
OLP-1002	Olipass	Na_v_1.7	Na_v_1.7 selective down-regulatory effect PNA	Phase 2	Currently in an Australian phase 2a clinical trial for treating osteoarthritis	[51,55]
QLS-278	Research Compound	Na_v_1.7 selective (TTX-sensitive)	Inactivation- and concentration-dependent Na_v_1.7 blockade; hyperpolarization shift of inactivation	Preclinical (2024)	IC_50_ 1.2 ± 0.2 μM; effective in neuropathic and inflammatory pain models	[56]
GDC-0276	Genentech/Roche	Na_v_1.7	Selective Na_v_1.7 voltage-gated sodium channel blockade	DISCONTINUED (Phase 1 terminated)	Dose-limiting hypotension and liver toxicity, discontinued in 2019	[57]
ST-2427	SiteOne (Lilly)	Na_v_1.7	-	DISCONTINUED (Phase 1 terminated)	Indication for pain, less information about clinical efficacy	[51,58,59]
iN1011-N17	iN Therapeutics	Na_v_1.7	Na_v_1.7 channel blocker	Phase 1 COMPLETED	Indication for pain, relatively safe in phase 1 trials.	[60,61]
PF-05089771	Pfizer	Na_v_1.7	Selective Na_v_1.7 sodium channel blockade	DISCONTINUED (Phase 2 failed)	Showed some efficacy in trigeminal neuralgia but failed broader neuropathic pain trials. Well-tolerated up to 450 mg bis in die	[62,63]
Vixotrigine (BIIB074/CNV1014802)	Biogen/Convergence	Na_v_1.7	Voltage- and use-dependent Na_v_1.7 channel blocker	DISCONTINUED (Phase 2/3 failed)	Developed for trigeminal neuralgia and peripheral neuropathic pain. Discontinued due to insufficient efficacy	[64,65,66]
Funapide (XEN402/TV-45070)	Xenon Pharmaceuticals	Na_v_1.7	Voltage-dependent Na_v_1.7 blockade (topical)	DISCONTINUED (Phase 2 failed)	Topical formulation for postherpetic neuralgia. Some responder subgroups identified, particularly Na_v_1.7 R1150W genetic carriers (63% vs. 35% response)	[67,68]
AZD-3161	AstraZeneca	Na_v_1.7	-	DISCONTINUED	Part of the Na_v_1.7 inhibitor failures; discontinued	[69]
PF-04531083	Pfizer	Na_v_1.7	Selective Na_v_1.7 sodium channel blockade	DISCONTINUED	Part of Pfizer’s Na_v_1.7 program alongside PF-05089771	[69]
**Multi-Target Na_v_ Channel Blockers**						
ANP-230/DSP-2230	Anest/Nippon Pharma	Na_v_1.7, Na_v_1.8, Na_v_1.9	Equipotent blockade of three pain-related Na_v_ channels; peripheral selectivity	Early Clinical Development	Innovative multi-target approach; addresses potential redundancy between Na_v_ subtypes	[70,71,72]
PnTx4(5-5)	Academic Research	Na_v_1.2–Na_v_1.6 + NMDA receptors	Dual mechanism-sodium channel blockade + NMDA autoreceptor modulation	Preclinical	*P. nigriventer* spider venom. More potent than MK-801; no motor impairment	[73]
ATX01	AlgoTherapeutix	Na_v_1.7, Na_v_1.8 and Na_v_1.9	Na_v_1.7, Na_v_1.8, Na_v_1.9 triple blocker	Phase 2 COMPLETED	Indicated for CIPN, locally delivered to the nerve endings where pain signals originate and propagate	[74]
Tetrodotoxin (TTX)	WEX Pharmaceuticals	Non-selective VGSC (especially Na_v_1.7, Na_v_1.8)	Highly specific voltage-gated sodium channel blocker; peripherally acting	Phase 2 COMPLETED	Positive trends in chemotherapy-induced neuropathic pain; natural neurotoxin from pufferfish	[75,76,77]
**Engineered Peptides & Biotechnology**						
Pro[LPATG_6_]Sx	Academic Research	Na_v_1.7 dual-site	Bivalent design: ProTx-II + SxIIIC fusion (gating modifier + pore blocker)	Research Tool	Engineering innovation: Sortase A ligation, combined mechanisms	[78]
PaurTx3	Academic Research	Na_v_1.2 and Na_v_1.7	Blockade by inducing a depolarizing shift in gating kinetics	Research Tool	Tarantula *Phrixotrichus auratus* venom. PaurTx3 exhibits a slower inhibition on Na_v_1.7 compared to Na_v_1.4 and Na_v_1.5	[78,79,80]
PnAn13	Academic Research	Multi-target	δ-Ctenitoxin derivative with cannabinoid system involvement	Preclinical	Synthetic optimization of spider toxin, multiple pain models	[81]
**Approved Agents**						
Lidocaine	Multiple (Generic)	Non-selective VGSC (Na_v_1.1–Na_v_1.9)	Use-dependent sodium channel blockade; prevents action potential propagation	FDA Approved	Gold standard for local and neuropathic pain; topical patches for PHN, IV infusion	[82,83,84]
Carbamazepine	Multiple (Generic)	Na_v_1.2, Na_v_1.3, Na_v_1.6 primarily	Use-dependent sodium channel blockade; stabilizes inactivated state	FDA Approved	First-line for trigeminal neuralgia; well-established efficacy	[84,85]
Oxcarbazepine	Multiple (Generic)	Na_v_1.2, Na_v_1.3, Na_v_1.6 primarily	Similar to carbamazepine but better tolerability profile	FDA Approved	Better tolerability than carbamazepine; second-generation anticonvulsant	[84,85]
Lacosamide	UCB/Pfizer	Non-selective VGSC	Slow inactivation enhancement (vs. fast inactivation blockade)	FDA Approved	Unique mechanism. Approved for epilepsy, off-label neuropathic pain. Cardiac monitoring required	[86,87]
Mexiletine	Multiple (Generic)	Na_v_1.5 (cardiac) + peripheral Na_v_s	Class IB antiarrhythmic; use-dependent sodium channel blockade	FDA Approved	Limited efficacy in pain studies; off-label pain use; significant side effects; originally antiarrhythmic	[88,89]
Dilantin/Phenytoin	Multiple (Generic)	Multiple VGSC subtypes	Voltage-dependent sodium channel blockade; multiple mechanisms	FDA Approved	Some evidence for acute zoster pain (IV fosphenytoin); limited chronic pain applications	[90,91]
Bupivacaine	Multiple (Pfizer Inc., Marcaine^®^ and Generic)	Non-selective VGSC (Na_v_1.1–Na_v_1.9)	Direct Nav blocker for regional anesthesia	FDA Approved	Canonical anesthesia. Blocks nerve impulses by increasing the threshold for nerve excitation, preventing pain signals from reaching the brain. Adverse effects (e.g., cardiotoxicity) linked to cardiac Na_v_1.5 inhibition	[92,93,94]
Ropivacaine	Glenmark Pharmaceuticals Inc. (Generic), AstraZeneca (Naropin)	Broad Na_v_ block (Na_v_1.1–Na_v_1.9)	Non-selective Nav blocker, similar site as bupivacaine with lower affinity for cardiac Na_v_1.5	FDA Approved	Canonical anesthetic Na_v_ blocker with reduced cardiotoxicity compared to bupivacaine	[95,96]
Amitriptyline	Multiple (original: Merck & Co, Elavil^®^)	Non-selective VGSC (Na_v_1.1–Na_v_1.9)	Use-dependent block of Na_v_ channels in addition to potent SERT/NET inhibition	FDA Approved	Canonical anesthesia. Indirect cardiac effects via multiple channels	[97,98,99]

CIPN, chemotherapy-induced peripheral neuropathy; FDA, Food and Drug Administration; IV, intravenous; NDA, new drug application; NMDA receptor, N-methyl-D-aspartate receptor; PHN, postherpetic neuralgia; PNA, peptide nucleic acid; VGSC, voltage-gated sodium channel; -, not clear or poorly investigated.

## Data Availability

No new data were created or analyzed in this study. Data sharing is not applicable to this article.

## Abbreviations

ATFB10	Familial atrial fibrillation, type 10
ATRST1	Atrial standstill, type 1
BFIS3	Benign familial infantile seizures, type 3
BRGDA1	Brugada syndrome, type 1
CIP	Congenital insensitivity to pain
CIPN	Chemotherapy-induced peripheral neuropathy
CMS16	Congenital myasthenic syndrome, type 16
CMD1E	Dilated cardiomyopathy, type 1E
CNS	Central nervous system
CPE	Cryptogenic partial epilepsy
DEKA	Asp–Glu–Lys–Ala
DRG	Dorsal root ganglia
DS	Dravet syndrome
EIEE6	Epileptic encephalopathy, early infantile, 6
EIEE11	Epileptic encephalopathy, early infantile, 11
EIEE13	Epileptic encephalopathy, early infantile, 13
FEB	Febrile seizures
FEB3A	Familial febrile seizures, type 3A
FEPS3	Familial episodic pain syndrome, type 3
FHM3	Familial hemiplegic migraine, type 3
FDA	Food and Drug Administration
GEFS+2	Generalized epilepsy with febrile seizures plus 2
GOF	Gain-of-function
GMTs	Gating-modifier toxins
HSAN7	Hereditary sensory and autonomic neuropathy, type 7
HOKPP2	Periodic paralysis, hypokalemic, type 2
HYPP	Periodic paralysis, hyperkalemic
IBD	Inflammatory bowel disease
IEM	Inherited erythromelalgia
ICEGTC	Intractable childhood epilepsy with generalized tonic–clonic seizures
IFM	Ile–Phe–Met
IV	Intravenous
LQT3	Long QT syndrome, type 3
LOF	Loss-of-function
MEPPC	Multifocal ectopic Purkinje-related premature contraction
MYOSCN4A	Myotonia SCN4A-related
NDA	New drug application
NMDA receptor	N-methyl-D-aspartate receptor
NSAIDs	Non-steroidal anti-inflammatory drugs
NKPP	Periodic paralysis, normokalemic
PD	Pore domain
PEPD	Paroxysmal extreme pain disorder
PFHB1A	Progressive familial heart block, type 1A
PHN	Postherpetic neuralgia
PNA	Peptide nucleic acid
PMC	Paramyotonia congenita of von Eulenburg
PKC	Protein kinase C
PNS	Peripheral nervous system
ProTx-II	Protoxin II
SFN	Small fiber neuropathy
SIDS	Sudden infant death syndrome
SSS1	Sick sinus syndrome, type 1
STX	Saxitoxin
TTX	Tetrodotoxin
VF1	Familial paroxysmal ventricular fibrillation, type 1
VGSC	Voltage-gated sodium channel
VSD	Voltage-sensing domain
